# Efficacy and safety of trans-sub-Tenon's retrobulbar anesthesia for pars plana vitrectomy: a randomized trial

**DOI:** 10.1186/s12886-022-02507-7

**Published:** 2022-06-30

**Authors:** Qian Xu, Meiqing Ren, Juanjuan Guan, Guihong Shi, Yan Ni, Jie Luan

**Affiliations:** 1 Department of Ophthalmology, Zhongda Hospital, Southeast University, Nanjing, 210009 Jiangsu Province China; 2 School of Medicine, Southeast University, Nanjing, 210009 China

**Keywords:** Retrobulbar anesthesia, Sub-Tenon anesthesia, Vitrectomy surgery

## Abstract

**Aim:**

To compare the efficacy and safety of trans-sub-Tenon's ciliary nerve block anesthesia and transcutaneous retrobulbar anesthesia in patients undergoing pars plana vitrectomy (PPV).

**Methods:**

A prospective, randomized, double-blinded clinical trial was conducted at Zhongda Hospital, Affiliated with Southeast University, from February 2021 to October 2021. Patients undergoing PPV were randomly allocated into two groups: the trans-sub-Tenon's anesthesia group (ST group) and the retrobulbar anesthesia group (RB group) in the ratio of 1:1. The ST group received 2 ml ropivacaine through the Tenon capsule to the retrobulbar space, while the RB group received 2 ml ropivacaine via transcutaneous retrobulbar injection. Visual analog score (VAS) was used to evaluate pain during the whole process, including during anesthesia implementation, intraoperatively and on the first day after the operation. Movement evaluation (Brahma scores) and anesthesia-related complications were also noted.

**Results:**

Finally, a total of 120 patients were included in the study (60 in the ST group and 60 in the RB group). There were no significant differences in baseline patient characteristics or surgical features between the two groups. The VAS pain scores for anesthesia implementation were 0.52 ± 0.47 in the ST group and 1.83 ± 0.87 in the RB group (*P* < 0.001). The VAS scores during the operation were 0.53 ± 0.49 in the ST group and 1.48 ± 1.02 in the RB group (*P* < 0.001) and those on the first day after the operation were 0.37 ± 0.38 in the ST group and 0.81 ± 0.80 in the RB group (*P* = 0.002). No patients required supplemental intravenous anesthesia intraoperatively. The Brahma movement scores were 0.70 ± 1.64 in the ST group (scores ranging from 0 to 8) and 2.38 ± 3.15 in the RB group (ranging from 0 to 12) (*P* = 0.001). Forty-two patients in each group received laser photocoagulation during surgery. Fifteen patients (36%) in the ST group could not see the flashes of the laser, compared to 8 patients (19%) in the RB group (*P* = 0.087). No serious sight-threatening or life-threatening complications related to anesthesia were observed in either group.

**Conclusions:**

For PPV, trans-sub-Tenon's ciliary nerve block anesthesia was more effective in controlling pain than transcutaneous retrobulbar anesthesia during the whole surgery process, including during anesthesia implementation, intraoperatively and on the first day after the operation. Additionally, it could achieve better effect of akinesia and was relatively safe. Trans-sub-Tenon's anesthesia could be considered an alternative form of local anesthesia during vitreoretinal procedures.

**Trial registration:**

The study protocol has been registered at ChiCTR.org.cn on February 2021 under the number ChiCTR2100043109.

**Supplementary Information:**

The online version contains supplementary material available at 10.1186/s12886-022-02507-7.

## Introduction

Most ophthalmic operations are performed under local anesthesia [1–3]. Surgery involving the eyeball requires ciliary nerve block anesthesia to relieve the patients' intraoperative pain. The literature [4–8] shows that the main local anesthesia methods of ciliary nerve include retrobulbar anesthesia and peribulbar anesthesia. However, peribulbar anesthesia cannot be accurately released near its action position, and its efficacy depends on the diffusion degree of anesthetic drugs in periocular tissues. If there is not enough anesthetic to reach the retrobulbar space, a satisfactory analgesic effect may not be obtained. Anesthesia for pars plana vitrectomy (PPV) is challenging because the surgery is usually longer than cataract surgery. The sharp long needle used in retrobulbar anesthesia could cause serious complications such as retrobulbar hemorrhage, ocular perforation or optic nerve injury. The incidence of these risks is higher, especially in patients with high myopia and posterior scleral staphyloma, during retrobulbar injection [9–11].

Tenon's capsule is a layer of dense fibrous tissue located around the eye that originates from the limbus and extends backwards to the optic nerve. The local anesthetic can be injected into the potential cavity between Tenon's capsule and posterior sclera and then diffuses into the retrobulbar space and around the sclera. The anaesthetic blocks both the sensory and motor nerves in this location to achieve ocular analgesia and dyskinesia [12]. Tenon's capsule anesthesia includes trans-sub-Tenon's retrobulbar anesthesia or peribulbar anesthesia (or localized sub-Tenon’s anesthesia applied to trocar entry sites [13]). The blocking of the ciliary nerve can be achieved after injecting anesthetic into the retrobulbar space via Tenon’s capsule. Compared with transcutaneous retrobulbar anesthesia that requires a sharp long needle, trans-sub-Tenon's retrobulbar anesthesia uses a blunt curved cannula, which reduces the possibility of serious complications such as eyeball perforation or optic nerve injury. It has been reported that trans-sub-Tenon's retrobulbar anesthesia can provide effective anesthesia for vitreoretinal surgery and reduces the risks associated with sharp needles [14–19]. However, some rare complications related to sub-Tenon injection have also been reported, including scleral perforation and retinal ischemia, especially in patients having previous ophthalmic surgery with conjunctival scarring and scleral thinning [20–23].

At present, there are few comparative clinical studies on trans-sub-Tenon's ciliary nerve block anesthesia and transcutaneous retrobulbar anesthesia for vitreoretinal surgery. There is no sufficient evidence whether trans-sub-Tenon's ciliary nerve block anesthesia is superior to or can replace transcutaneous retrobulbar anesthesia. Therefore, our study prospectively compared the effectiveness and safety of trans-sub-Tenon's ciliary nerve block anesthesia and transcutaneous retrobulbar anesthesia in patients undergoing PPV to provide evidence for the selection of anesthesia methods.

## Methods

This prospective, randomized, double-blinded clinical trial was conducted at Zhongda Hospital, Affiliated with Southeast University, from February 2021 to October 2021. The study adhered to the tenets of the Declaration of Helsinki and was approved by the Clinical research ethics committee of Zhongda Hospital, Affiliated with Southeast University, on December 31st, 2020. All possible risks and benefits were explained to the eligible patients before they were enrolled in the study, and informed consent was obtained before any treatment was given. The study protocol has been registered at ChiCTR.org.cn under the number ChiCTR2100043109.

The criteria for admission included: (1) patients admitted to the hospital for PPV surgery; (2) the age was from 18 to 80 years old; (3) patients who could tolerate vitreoretinal surgery after excluding surgical contraindications; (4) patients voluntarily participated in the clinical trial and signed informed consent. Exclusion criteria: (1) history of previous vitreoretinal surgery; (2) history of ocular trauma; (3) the patient could not cooperate with the operation; (4) the patient was allergic to ropivacaine or any of its components or similar drugs; (5) the patient was pregnant or lactating female. The sample size was calculated based on previous literature review [13, 17, 24]. The estimated sample size was 120 patients (60 in each group) based on type I error (α) of 5%, and a power (1-β) of 90%.

The patients agreed to participate and signed the informed consent form. They were then randomly allocated into two groups: the trans-sub-Tenon's ciliary nerve block anesthesia group (ST group) and the transcutaneous retrobulbar anesthesia group (RB group) in the ratio of 1:1 according to randomization list. Patients were masked to the type of anesthesia received. Before the surgery, each patient underwent a detailed eye examination and routine laboratory investigations. About 5 min before anesthesia, patients were provided with appropriate information about the anesthesia and surgery to reduce their anxiety. However, patients did not know the type of anesthesia they received. In addition, all patients were given topical anesthetic with oxybuprocaine hydrochloride eye drops for the operated eye every 5 min for a total of four times before the surgery. All patients inhaled continuous oxygen at 2–3 L/min via nasal oxygen cannula during the operation.

Patients assigned to the transcutaneous retrobulbar anesthesia group (RB group) received 2 ml of 0.75% ropivacaine for retrobulbar anesthesia. The transcutaneous retrobulbar injection was conducted by inserting a sharp retrobulbar needle through the lower eyelid and orbital septum at the junction of the lateral and middle thirds of the inferior orbital rim and into the retrobulbar space. Patients assigned to the trans-sub-Tenon's ciliary nerve block anesthesia group (ST group) received 2 ml 0.75% ropivacaine through Tenon’s capsule to the retrobulbar space. An inferonasal small incision was made through the conjunctiva and Tenon’s tissue with scissors to expose the sclera surface. A blunt sub-Tenon’s cannula was then inserted into the posterior sub-Tenon’s space, and 2 mL solution of ropivacaine was then injected slowly with the blunt curved cannula. Two minutes later, the patients underwent standard 23-gauge pars plana vitrectomy, and all operations were performed by one experienced retina surgeon (Dr Jie Luan). If the patient felt pain during the operation, supplemental anesthesia was conducted with the injection of 2 ml ropivacaine via sub-Tenon’s space.

### Pain evaluation

A researcher who was not involved in the surgery and anesthesia protocols used visual analog score (VAS, 0–10, 0 = no pain and 10 = worst pain imaginable) to evaluate the pain felt by the patient during the whole process (including during anesthesia implementation, intraoperatively and on the first day after the operation). And the researcher was also masked to the type of anesthesia used.

### Movement evaluation

Extraocular motility (Brahma score) was used to assess superior, inferior, medial, and lateral movement (scores: 3 = normal, 2 = partial movement, 1 = almost no movement, and 0 = no movement) [25].

### Subconjunctival hemorrhage and chemosis evaluation

Chemosis and subconjunctival hemorrhage each received a score and were graded as follows: 2 = completely obscuring surgery, 1 = mildly obscuring surgery, and 0 = not obscuring surgery [26, 27].

Additionally, patients treated with retinal photocoagulation were asked whether they could see the flashes of the laser during the process. Any anesthetic-related complications, such as retrobulbar hemorrhage, globe perforation, optic nerve injury, brainstem anesthesia and so on, were also noted.

### Statistics

The results were presented as the mean ± standard deviation (SD) for continuous variables. The t-test was used for normally distributed data; otherwise, the Mann–Whitney U test was used. Categorical variables were presented as the number of patients (n), and comparisons between the two groups were performed using Chi-square test. SPSS 20.0 was used for statistical analysis, and a *P* value < 0.05 was considered statistically significant.

## Results

Finally, a total of 120 patients were included in the study (60 in the ST group and 60 in the RB group) between January 2021 and October 2021. Baseline patient characteristics and surgical features of each group were summarized in Table 1. There were no significant differences between the groups with respect to age, gender, comorbidities (diabetes mellitus, hypertension, cardiovascular or cerebrovascular disease, chronic kidney disease), surgical indication (vitreous hemorrhage, retinal detachment or macular diseases), type of surgery (Phaco + PPV or PPV), intraoperative photocoagulation treatment, cryotherapy treatment and duration of surgery. Forty-two patients in each group received laser photocoagulation during the surgery. Fifteen patients (36%) in the ST group could not see the flashes of the laser, compared to 8 patients (19%) in the RB group (*P* = 0.087).

**Table 1:** Baseline patient characteristics and surgical features

	Sub-Tenon (ST) Group	Retrobulbar (RB) Group	*P* value
Age (years, mean ± SD)	58 ± 10	57 ± 11	0.352*
Male/Female (n)	29/31	34/26	0.361**
Comorbidities			
Diabetes mellitus (n)	24	26	0.711**
Hypertension (n)	35	29	0.272**
Cardiovascular or cerebrovascular disease (n)	11	8	0.453**
Chronic kidney disease (n)	6	4	0.509**
Surgical indication			0.808***
Vitreous hemorrhage (n)	30	33	
Retinal detachment (n)	22	22	
Macular disease (n)	7	4	
Others (n)	1	1	
Type of surgery			
Phaco + PPV/PPV (n)	37/23	32/28	0.356**
Endolaser photocoagulation (n)	42	42	
Could not see the laser (n)	15	8	0.087**
Cryotherapy (n)	15	22	0.166**
Duration of surgery (minutes, mean ± SD)	60.6 ± 23.3	57.8 ± 21.5	0.426*

^*^ Wilcoxon; ** Pearson; *** Likelihood Ratio

*n* number of patients, *SD* Standard deviation

### Pain evaluation

**Fig. 1:**
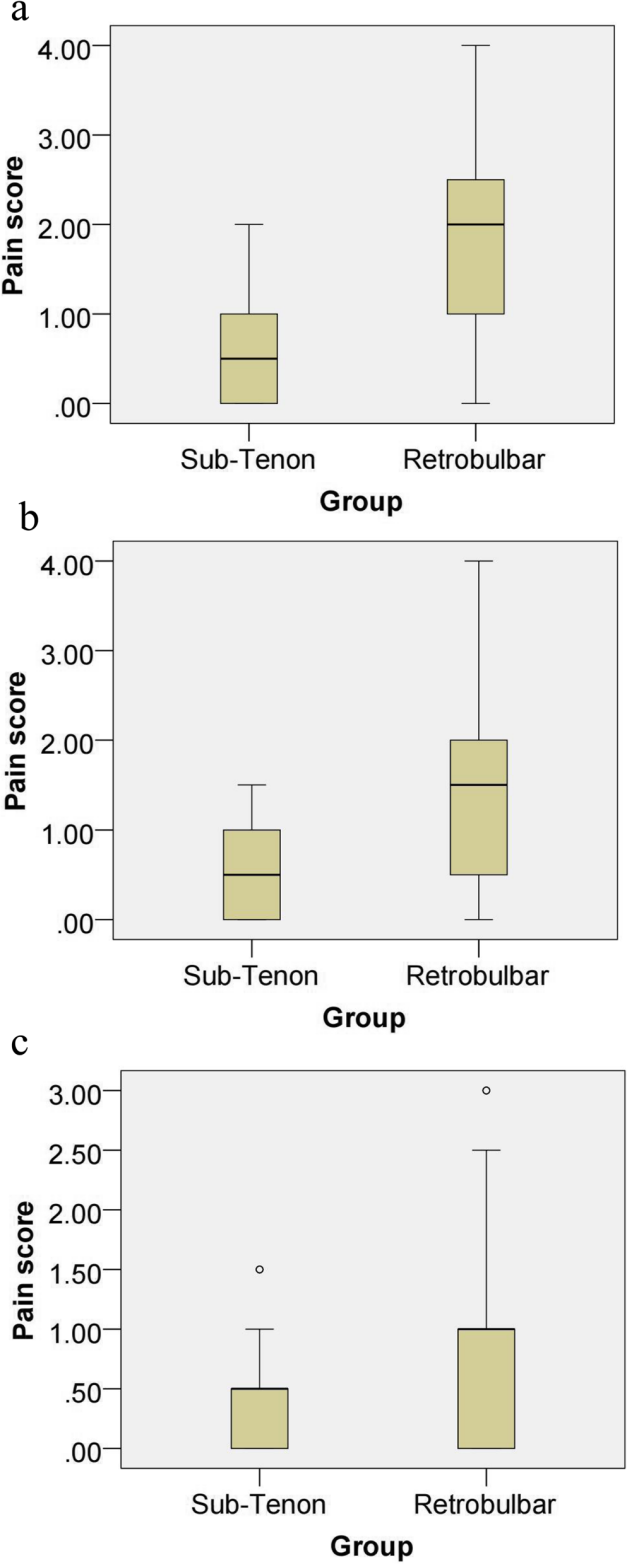
VAS pain scores of the two groups during the whole process, including during anesthesia implementation (**a**), intraoperatively (**b**) and on the first day after the operation (**c**)

Table 2 showed the VAS pain scores of the two groups during the whole process. The mean VAS pain scores during anesthesia implementation were 0.52 ± 0.47 in the ST group and 1.83 ± 0.87 in the RB group (*P* < 0.001) (Fig. 1a). The mean VAS pain scores during the operation were 0.53 ± 0.49 in the ST group and 1.48 ± 1.02 in the RB group (*P* < 0.001) (Fig. 1b) and those on the first day after the operation were 0.37 ± 0.38 in the ST group and 0.81 ± 0.80 in the RB group (P = 0.002) (Fig. 1c). Seven of the 60 patients from the ST group rated their pain level as zero during the whole process (during anesthesia implementation, intraoperatively and on the first day after the operation), meaning that they experienced no pain at all. Additionally, the maximum pain score in the ST group was 2. In the RB group, only 1 of the 60 patients rated their pain level as a zero during the whole process, and the maximum pain score was 4. Thus, during the whole process of anesthesia implementation, surgery and the first day after operation, the patients in the ST group experienced significantly less pain than the patients in the RB group.

**Table 2:** VAS pain and Brahma movement scores

	Sub-Tenon (ST) Group	Retrobulbar (RB) Group	*P*
VAS scores			
Anesthesia implementation	0.52 ± 0.47	1.83 ± 0.87	< 0.001*
Intraoperatively	0.53 ± 0.49	1.48 ± 1.02	< 0.001*
On the first day after the operation	0.37 ± 0.38	0.81 ± 0.80	0.002*
Brahma movement scores	0.70 ± 1.64	2.38 ± 3.15	0.001*

^*^ Wilcoxon

No patients needed supplemental intravenous anesthesia intraoperatively. Four patients in the ST group felt pain and then received supplemental local anesthesia intraoperatively, compared to 56 patients in the RB group (*P* < 0.001) (Table 3). Among them, 3 patients in the ST group felt pain during placement of the trocar-cannula system, compared to 43 patients in the RB group (*P* < 0.001). One patient in the ST group felt pain during scleral depression, compared to 13 patients in the RB group (*P* = 0.001). The pain levels during scleral cannula insertion and scleral depression were higher in the RB group than in the ST group.

**Table 3:** Supplemental local anesthesia intraoperatively

	Sub-Tenon (ST) Group	Retrobulbar (RB) Group	*P*
During placement of the trocar-cannula system (n)	3	43	< 0.001**
During scleral depression (n)	1	13	0.001**
Supplemental local anesthesia (n)	4	56	< 0.001**

^**^ Pearson

### Brahma movement scores

The mean Brahma movement scores were 0.70 ± 1.64 in the ST group (scores ranging from 0 to 8) and 2.38 ± 3.15 in the RB group (ranging from 0 to 12) (*P* = 0.001) (Table 2). Akinesia was 82% (49 patients) in the ST group and 55% (33 patients) in the RB group. In the RB group, one patient lacked intraoperative akinesia and had normal eye movements during the operation, which achieved 12 scores.

### Subconjunctival hemorrhage and chemosis scores

Subconjunctival hemorrhage obscuring the surgical view was rare; only one case in the ST group had a hemorrhage obscuring some of the surgical view, which was score as a 1, and the rest of the cases were scored as 0. All patients in the two groups achieved scores of 0 on the chemosis evaluation.

In addition, no serious sight-threatening complications (such as retrobulbar hemorrhage, globe perforation, or optic nerve injury) or life-threatening complications (such as brainstem anesthesia) related to anesthesia were observed in either group.

## Discussion

The main local anesthesia methods for the PPV operation include sub-Tenon's anesthesia, retrobulbar anesthesia and peribulbar anesthesia. The efficacy of peribulbar anesthesia depends on the diffusion degree of the anesthetic drugs in periocular tissues. If there is not enough anesthetic to reach the retrobulbar space, satisfactory analgesic effect may not be obtained. Therefore, to ensure that a sufficient amount of anesthetic reaches the retrobulbar space, peribulbar anesthesia requires a larger volume of anesthetic (up to 10 ml) with or without hylase to help spread the anesthetic [28–30]. Moreover, a randomized trial from 2020 comparing sub-Tenon's anesthesia (2–4 ml ropivacaine) and peribulbar anesthesia (4–6 ml ropivacaine) for PPV showed that sub-Tenon's anesthesia was more effective in controlling pain during the whole vitrectomy procedure than peribulbar anesthesia [24]. To date, there were few comparative clinical studies on the trans-sub-Tenon's ciliary nerve block anesthesia and transcutaneous retrobulbar anesthesia for vitreoretinal surgery. Two comparative articles [17, 31] were retrieved, and there were some differences between the studies. A prospective controlled study [17] from the United States in 2004 (30 eyes via trans-sub-Tenon's ciliary nerve block anesthesia and 34 eyes by transcutaneous retrobulbar anesthesia) compared two methods of anesthesia with the auxiliary preparation of intravenous anesthetic sedation. The results showed that trans-sub-Tenon's anesthesia was as effective as transcutaneous retrobulbar anesthesia in controlling intraoperative pain. However, the use of preoperative intravenous sedatives might have some impact on the analyses. A retrospective comparative study [31] from the United States in 2016 (938 eyes were anesthetized by sub-Tenon's ciliary nerve block and 770 eyes by transcutaneous retrobulbar anesthesia) showed that trans-sub-Tenon's anesthesia was as effective and safe as transcutaneous retrobulbar anesthesia for PPV. The rates of conversion to general anesthesia were low (9 cases and 12 cases, respectively), and the reasons were similar, including patient anxiety and discomfort. The authors suggested that surgeons might wish to consider using sub-Tenon's anesthesia for local anesthesia during vitreoretinal procedures. Nevertheless, this large sample size study also had some limitations, including the difference of surgeons (the operations were performed by five different surgeons) and bias of the surgeons in the choice of anesthesia, which might have effects on the result.

Therefore, different from previous comparative studies, our research made the following improvements. First, this was a prospective, randomized, double-blind clinical trial that directly compared the efficiency and safety of trans-sub-Tenon's ciliary nerve block anesthesia and transcutaneous retrobulbar anesthesia in pars plana vitrectomy. Second, all patients in our study received the same amount of local anesthetic without intravenous anesthesia sedation, which controlled the confounding factors to some extent. Sub-Tenon's anesthesia included anterior or posterior injection techniques. Anterior injections were performed superficially just beyond the equator with a low volume (3 to 5 ml) of anesthetic. In contrast, posterior injections could provide more anesthetic into the posterior spaces, which allowed for a smaller volume of anesthetic and thus a lower risk of chemosis [1, 12–19, 27, 32]. We delivered 2 ml ropivacaine directly into the posterior space via a posterior sub-Tenon injection. Third, for patients undergoing PPV in our study, the surgical indications mainly included vitreous hemorrhage caused by various reasons, retinal detachment and macular diseases. Intraoperative treatments contained photocoagulation and cryotherapy. There were no statistical difference of baseline patient characteristics or surgical features between groups, enabling a better comparison of the two anesthesia methods. Fourth, our study used ropivacaine as a local anesthetic in both groups. In the previous two studies [17, 31], the local anesthetic used was an equal mixture of lidocaine and bupivacaine. A prospective randomized comparative study [33] in 2017 showed that ropivacaine alone could provide sufficient intraoperative anesthesia similar to bupivacaine, lidocaine or lidocaine + bupivacaine mixture and had better postoperative analgesic effect with lower occurrence of postoperative subconjunctival hemorrhage. The commonly used ophthalmic local anesthetic drugs included lidocaine, ropivacaine and bupivacaine. The studies [33–37] indicated that three drugs were all effective for ophthalmic local anesthesia. Lidocaine had the characteristics of fast onset and short duration of action. Bupivacaine and ropivacaine had similar efficacy, while ropivacaine might cause less cardiovascular effect than bupivacaine. It was suggested that ropivacaine might become the drug of choice for eye block anesthesia because of its duration of action and safety. Fifth, we added evaluation of intraoperative light perception loss for the two anesthesia methods. Patients treated with retinal photocoagulation were asked whether they could see the flashes of the laser during the process. The results suggested that 36% of patients who received sub-Tenon anesthesia could not see the laser during the operation, compared with 19% who received transcutaneous retrobulbar anesthesia. Anesthesia-induced temporary conduction block of optic nerve was considered a reasonable explanation for this difference [38]. Since anesthetic via transcutaneous retrobulbar injection could not always reach the muscle pyramid near the optic nerve, this technique might have less occurrence of intraoperative light perception loss. Sixth, we recorded the steps of discomfort or pain during the operation and compared the two groups. At the beginning of vitreoretinal surgery, the insertion of trocar might make the patient feel pain (3 patients in the ST group compared to 43 patients in the RB group, P < 0.001). Although the pain might be sudden and short term, it might cause anxiety and fear in patients and affect the comfort of patients and surgeons [13]. In addition, to observe and deal with peripheral retinopathy, it was necessary to press the sclera during vitreoretinal surgery, which would also lead to discomfort (ST group, one patient; RB group, 13 patients, P = 0.001). These observations showed that the pain levels with transcutaneous retrobulbar anesthesia were greater during scleral cannula insertion and scleral depression than those with trans-sub-Tenon's anesthesia.

Finally, our results suggested that with the use of the same low volume of anesthetic (2 ml ropivacaine) in both groups, patients who received trans-sub-Tenon's ciliary nerve block anesthesia experienced less pain than patients with transcutaneous retrobulbar anesthesia during the whole 23-gauge PPV surgery process, including during anesthesia implementation, intraoperatively and on the first day after the operation. The sub-Tenon technique could get better effect of akinesia. Compared with the same amount of transcutaneous retrobulbar anesthesia, trans-sub-Tenon's ciliary nerve block directly provided anesthesia to the sclera and ciliary nerve by inserting blunt sub-Tenon’s cannula into the posterior sub-Tenon’s space and could be more effective. In addition, anesthesia-related complications were not observed in either group of our study, and both anesthesia techniques were relatively safe. However, studies showed that blunt sub-Tenon’s cannula was inherently safer than sharp needle [21]. It could reduce the incidence of needle-related complications, especially for patients with high myopia and posterior scleral staphyloma. In previous reports, the sharp needle for local retrobulbar or peribulbar anesthesia was associated with a 2.5-fold increase in serious eye complications over blunt cannulas [21]. Moreover, brainstem anesthesia leading to serious neurological complications was observed during retrobulbar or peribulbar anesthesia with sharp needle but not seen with trans-sub-Tenon’s anesthesia[39]. However, due to the rare incidence of complications, our study was not powered to detect a difference between the two techniques, and it was a limitation of this study. A larger sample size may even require several more thousand cases to show significant difference in these rare complications. Additionally, the recall bias of transient pain might also be one of the limitations of the study. The mean pain scores for both techniques were below 3. Pain below 3 was typically tolerated very well. However, 56 patients undergoing transcutaneous retrobulbar anesthesia felt transient pain during the surgical process (43 patients during trocar insertion, 13 patients during scleral depression). Although the pain might be sudden and short term, we immediately stopped the ongoing operation and provided timely supplemental local anesthetic. Four patients with sub-Tenon's anesthesia felt pain, indicating that sub-Tenon's anesthesia was the better option and was clinically acceptable under low-dose anesthesia, while 2 ml of anesthetic was not enough for transcutaneous retrobulbar anesthesia. In addition, due to the timely supplementation of anesthesia, the transient pain was relieved. At the end of the operation, when patients were asked to recall their pain during the whole process, the scores might be low because of recall bias. Moreover, PPV combined with buckling was also one method of vitreoretinal surgery. However, buckling might cause more discomfort and pain because of the traction on the extraocular muscles during the process which could influence the pain scores. No buckling patients were included in our study and it might be another limitation.

## Conclusions

For patients undergoing pars plana vitrectomy, trans-sub-Tenon's ciliary nerve block anesthesia was more effective in controlling pain than transcutaneous retrobulbar anesthesia during the whole surgery process, including during anesthesia implementation, intraoperatively and on the first day after the operation. Additionally, it could achieve better effect of akinesia and was relatively safe. Trans-sub-Tenon's ciliary nerve block anesthesia could be considered an alternative form of local anesthesia during vitreoretinal procedures.

## Supplementary Information

**Additional file 1.**

## Data Availability

The datasets generated and/or analyzed during the current study are included in this published article and its supplementary information files.
